# Platelet-rich plasma enhances the proliferation of human adipose stem cells through multiple signaling pathways

**DOI:** 10.1186/s13287-018-0851-z

**Published:** 2018-04-16

**Authors:** Fangyuan Lai, Natsuko Kakudo, Naoki Morimoto, Shigeru Taketani, Tomoya Hara, Takeshi Ogawa, Kenji Kusumoto

**Affiliations:** 1 Department of Plastic and Reconstructive Surgery, Kansai Medical University, 2-5-1 Shin-machi, Hirakata, Osaka, 573-1010 Japan; 2 Department of Microbiology, Kansai Medical University, Osaka, 573-1010 Japan; 3 Department of Oral Implantology, Osaka Dental University, Osaka, 573-1121 Japan

## Abstract

**Background:**

Platelet-rich plasma (PRP) is an autologous blood product that contains a high concentration of several growth factors. Platelet-derived growth factor (PDGF)-BB is a potential mitogen for human adipose-derived stem cells (hASCs). PRP stimulates proliferation of hASCs; however, the signaling pathways activated by PRP remain unclear.

**Methods:**

hASCs were cultured with or without PRP or PDGF-BB, and proliferation was assessed. hASCs were also treated with PRP or PDGF-BB with or without imatinib, which is a PDGF receptor tyrosine kinase inhibitor, or sorafenib, which is a multikinase inhibitor. Inhibition of cell proliferation was examined using anti-PDGF antibody (Abcam, Cambridge, UK), by cell counting. We assessed the effects of inhibitors of various protein kinases such as ERK1/2, JNK, p38, and Akt on the proliferation of hASCs.

**Results:**

The proliferation was remarkably promoted in cells treated with either 1% PRP or 10 ng/ml PDGF-BB, and both imatinib and sorafenib inhibited this proliferation. Anti-PDGF antibody (0.5 and 2 μg/ml) significantly decreased the proliferation of hASCs compared with control. PRP-mediated hASC proliferation was blocked by inhibitors of ERK1/2, Akt, and JNK, but not by an inhibitor of p38.

**Conclusions:**

PRP promotes hASC proliferation, and PDGF-BB in PRP plays a major role in inducing the proliferation of hASCs. PRP promotes hASC proliferation via ERK1/2, PI3K/Akt, and JNK signaling pathways.

## Background

Human adipose-derived stem cells (hASCs) were first isolated from human adipose tissue and identified by Zuk et al. in 2001 [1]. These cells can differentiate toward multiple lineages, such as osteogenic [2], chondrogenic [3], adipogenic [4], cardiac [5], epidermal [6], and neurogenic [7] lineages. hASCs are used widely in the field of regenerative medicine, including to promote bone regeneration [2], tooth and periodontal regeneration [8], cartilage regeneration [9], wound healing [6, 10], and nerve regeneration to cure Parkinson’s disease [11], as well as to suppress aging [10]. Due to the advantages of the autologous source of these cells and their relative abundance and ease of isolation, hASCs have also been widely used in the fields of plastic surgery and regenerative medicine [12].

However, the proliferation and differentiation capacities of hASCs decrease with age [13, 14], body mass index [14], diabetes mellitus [12, 15], radiation exposure [16], and tamoxifen treatment [17]. hASCs account for about 16–30% of the stromal vascular fraction [18]. To obtain a sufficient amount of cells for therapeutic purposes, in-vitro proliferation of the cells is required. Fetal bovine serum (FBS) is widely used for this purpose in multiple types of cells in vitro. However, due to the risk of heterologous immunization and zoonosis, FBS has limited clinical use.

Platelet-rich plasma (PRP) is a blood portion that is enriched with platelets [19]. Upon activation, platelets in PRP release granules containing molecules including growth factors and regulatory proteins, such as platelet-derived growth factor (PDGF), epidermal growth factor (EGF), insulin-like growth factors (IGFs), transforming growth factor beta (TGF-β), vascular endothelial growth factor (VEGF), and others [19–21]. These growth factors play important roles in cell proliferation, migration, and differentiation.

Our previous study revealed that activated PRP has a potential effect on the proliferation of hASCs and human dermal fibroblasts (hDFs) compared with nonactivated PRP [22]. Furthermore, we also reported that activated PRP induces hDF proliferation via the activation of ERK1/2 signaling [23]. Recently, other investigators reported that PDGF also enhances proliferation of hASCs through the JNK pathway [24]. However, the signaling pathways involved in PRP-stimulated proliferation of hASCs have not been clarified.

In the present study, we show that PRP stimulated cell proliferation by ERK1/2, JNK, and Akt activation. We compared this effect with the proliferative effect of PDGF-BB, a major growth factor in PRP.

## Methods

### Preparation of activated PRP

Activated PRP was obtained using the double-spin method as described previously [23]. Briefly, after obtaining informed consent from healthy adult volunteers (*n* = 3), blood was collected in tubes containing an acid-citrate-dextrose solution formula A anticoagulant, and spun in a standard laboratory centrifuge for 7 min at 450×*g*. The yellow plasma with buffy coat, containing platelets, leukocytes, and some erythrocytes from two tubes, was collected in a monovette via a long cannula and centrifuged for 5 min at 1600×*g*. Platelets that accumulated in the thrombocyte pellet in 1.0 ml plasma were used as PRP. A separate sample of 8 ml blood was allowed to stand for 30 min at room temperature in a tube without anticoagulant and then spun for 8 min at 2015×*g*. The supernatant was collected as an autologous thrombin. A 1:1 (v/v) mixture of 0.5 M CaCl_2_ and autologous thrombin was prepared in advance as an activator. A 10:1 (v/v) mixture of PRP and activator was incubated for 10 min at room temperature. Activated PRP was centrifuged at 90×*g* and then 9000×*g* for 10 min each; the supernatant was filtered through a 0.22-μm membrane (Millex GP; Merck Millipore, Tullagreen, Carrigtwohill, Co. Cork, Ireland) and stored at −80 °C until use.

### Measurement of platelet concentrations and growth factor levels

The number of platelets in whole plasma and PRP was counted using an XE-2100 automated hematology system (Sysmex Corp., Tokyo, Japan). PDGF-BB, IGF, and EGF levels in whole plasma and activated PRP were determined using commercially available ELISA kits (R&D Systems, Minneapolis, MN, USA), according to the manufacturer’s instructions.

### Isolation of hASCs

Unnecessary adipose tissue was obtained from a 61-year-old male patient who had previously provided informed consent and underwent plastic surgery. hASCs were isolated using a method described previously [25]. After washing extensively with phosphate-buffered saline (PBS), the adipose tissues were cut into small pieces and incubated with 3 volumes of 0.1% collagenase (Sigma-Aldrich, St. Louis, MO, USA) solution with constant shaking at 40 °C for 40 min. After adding DMEM containing 10% FBS (Hyclone, Logan, UT, USA) and antibiotics (complete medium), the tissue was centrifuged at 400×*g* for 3 min. After removing cellular debris through a 100-μm nylon mesh (BD Falcon, Bedford, MA, USA), the cells were incubated in complete medium in a dish. The primary hASCs were cultured for 4–5 days until they reached confluence. These cells were defined as passage “0”. For all experiments, cells from passages 7–9 were used.

### Cell proliferation assay

For the cell proliferation assays, hASCs were seeded at a density of 1.0 × 10^4^ cells/well in 24-well culture plates and incubated in complete medium overnight. The cell medium was then replaced with serum-free DMEM. After 6 h of incubation, hASCs were treated with PRP or human recombinant PDGF-BB (PeproTech EC Ltd, London, UK) at the stated concentrations in serum-free DMEM for 48 h. Inhibitors included the PDGF receptor tyrosine kinase inhibitor imatinib (Wako Co., Ltd, Tokyo, Japan), the multikinase inhibitor sorafenib (AdooQ, Irvine, CA, USA), the MEK inhibitor PD98059, the phosphatidylinositol-3-kinase-Akt inhibitor LY294002, the p38 inhibitor SB203580 (Calbiochem-Novabiochem, San Diego, CA, USA), and the JNK inhibitor SP600125 (Sigma). Inhibitors were added 1 h before the incubation with PRP or PDGF-BB. Cell proliferation was determined using Cell Counting Kit-8 (Dojindo Molecular Technologies, Kumamoto, Japan), according to the manufacturer’s instructions. Absorbance was read at 450 nm on a multiwell plate reader (EnSpire 2300 Multilabel Reader; PerkinElmer, Inc., Waltham, MA, USA).

To estimate the cell number from the absorbance, a standard curve was established. hASCs were seeded at densities of 0, 6250, 12,500, 25,000, 50,000, and 100,000 cells/well in 24-well plates for 3 h with 10% FBS in DMEM. The cells were then incubated with Cell Counting Kit-8 solution for 1 h, and the absorbance was read. The standard curve was established by plotting the number of cells versus the absorbance.

### BrdU incorporation

hASCs were seeded at a density of 2 × 10^3^ cells/well in 96-well culture plates containing complete medium. After overnight incubation, the hASCs were first starved in serum-free DMEM for 6 h and then treated with PRP, PDGF-BB, human recombinant IGF, or human recombinant EGF (PeproTech EC Ltd) in serum-free DMEM for 48 h. Inhibitors were added 1 h before incubation with PRP or PDGF-BB. Quantification of cell proliferation was determined using the Cell Proliferation ELISA BrdU kit (Roche, Mannheim, Germany).

### Cell cycle assay

hASCs (1 × 10^6^ cells) were seeded in a 10-cm culture dish containing complete medium and cultured overnight. The medium was then replaced with serum-free DMEM for 6 h, and the cells were treated with reagents at the stated concentrations for 48 h. Treated cells were collected by trypsinization. After washing with ice-cold PBS twice, cells were fixed in 70% ethanol at −20 °C for 3 h. The fixed cells were then stained with Muse™ Cell Cycle reagent (Millipore) in the dark at room temperature for 30 min. Cell cycle phases were analyzed by flow cytometric quantification of DNA with the Muse™ Cell Analyzer (Millipore).

### Western blot analysis

The cells were treated with the indicated compounds and lysed. Extracted cellular proteins (20 μg) were separated by sodium dodecyl sulfate-polyacrylamide gel electrophoresis (SDS-PAGE) and then transferred to a polyvinylidene difluoride (PVDF) membrane. The membrane was first blocked with Blocking One-P reagent (Nacalai Tesque, Kyoto, Japan) for 30 min at room temperature, and then incubated with the following primary antibodies: rabbit anti-phospho-ERK1/2 (1:1000; Epitomics Inc., Burlingame, CA, USA), rabbit anti-phospho-Akt, rabbit anti-Akt (1:5000; Abcam, Cambridge, UK), rabbit anti-ERK1/2 (1:1000; Cell Signaling Technology, Beverly, MA, USA), or rabbit anti-β-actin (1:1000; Cell Signaling Technology) at 4 °C overnight. This was followed by incubation with peroxidase-linked secondary antibody (1:20000; GE Healthcare, Little Chalfont, UK) at room temperature for 30 min. The labeled proteins were detected with enhanced chemiluminescence using the Prime Western blotting detection system (GE Healthcare).

### In-vitro JNK activity assay

Activity of the JNK pathway was analyzed with the SAPK/JNK Kinase Assay Kit (Cell Signaling Technology). Briefly, cell lysates were immunoprecipitated with the anti-phospho-JNK antibody coupled to JNK sepharose beads. The concentrated active JNK protein was then reacted with the substrate, c-Jun fusion protein, in the presence of ATP. The reaction mixture was separated with SDS-PAGE and transferred to a PDVF membrane. The membrane was incubated with rabbit anti-phospho-c-Jun (1:1000) at 4 °C overnight, followed by incubation with peroxidase-linked anti-rabbit IgG at room temperature for 30 min. The labeled proteins were also detected with enhanced chemiluminescence using the Prime Western blotting detection system. All of the experiments were replicated three times.

### Statistical analysis

Data are presented as the mean ± standard deviation (SD). The Mann–Whitney *U* test was used to evaluate differences among groups. *P* < 0.05 and *P* < 0.01 were considered statistically significant.

## Results

### Concentrations of platelet and growth factors in PRP and blood

Compared with whole plasma, PRP showed a 10.1-fold enrichment in platelets and a 25.9-fold enrichment in PDGF-BB. In contrast, the concentrations of EGF were comparable between PRP and whole plasma. IGF in PRP was present at a concentration of only 60% that in whole plasma (Table 1).

**Table 1:** Platelet concentrations and growth factor levels in serum and PRP (*n* = 3)

	Platelets (×10^10^/L)	PDGF-BB (ng/ml)	EGF (pg/ml)	IGF-I (ng/ml)
Plasma	15.5 ± 2.3	1.5 ± 0.4	534 ± 8	226 ± 6
PRP	157.5 ± 10.6	38.8 ± 0.7	639 ± 17	145 ± 4
Ratio (PRP/plasma)	10.1	25.9	1.2	0.6

*PDGF* platelet-derived growth factor, *EGF* epidermal growth factor, *IGF* insulin-like growth factor, *PRP* platelet-rich plasma

### PRP stimulated proliferation of hASCs

**Fig. 1:**
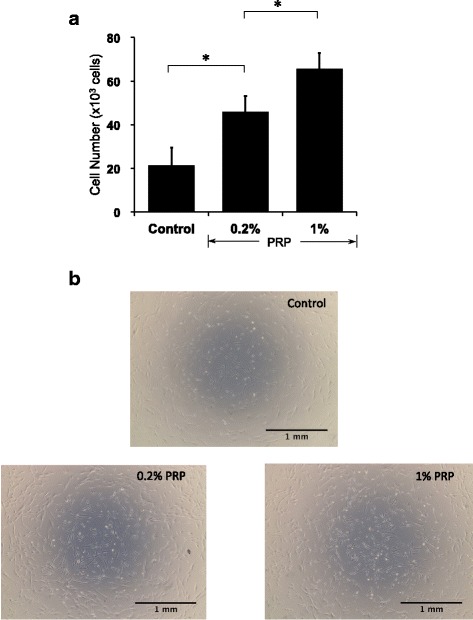
PRP stimulated proliferation of hASCs. Cells were incubated with PRP in serum-free DMEM for 48 h. Growth examined with Cell Counting Kit-8 by reading absorbance at 450 nm. **a** PRP stimulated hASC proliferation in a dose-dependent manner (*n* = 7). *P < 0.01. **b** Phase-contrast micrographs show increase in hASCs after treatment with PRP. PRP platelet-rich plasma

Cell proliferation was increased by treatment with 0.2% PRP (*P* < 0.01 vs control), and 1% PRP stimulated cell proliferation to a greater extent (*P* < 0.01 vs control and *P* < 0.01 vs 0.2% PRP). Thus, PRP stimulated proliferation of hASCs in a dose-dependent manner between 0 and 1% PRP (Fig. 1a). The proliferation was decreased when cells were treated with 3% and 5% PRP, compared with 1% (data not shown). The cell growth stimulated by PRP was confirmed by observation with phase-contrast microscopy (Fig. 1b).

### PRP containing PDGF-BB promoted hASC proliferation

**Fig. 2:**
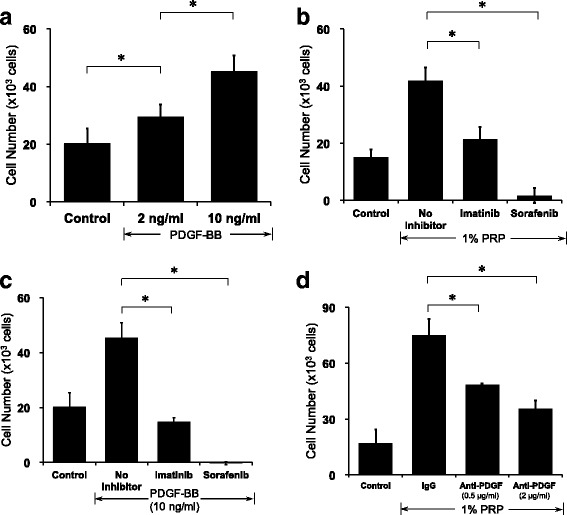
PDGF-BB mediated the stimulatory effect of PRP. Cells were incubated with reagents at indicated concentrations in serum-free DMEM for 48 h. Cell proliferation assessed with Cell Counting Kit-8. **a** PDGF-BB enhanced hASC proliferation in a dose-dependent manner (*n* = 7). **b** Effect of imatinib (5 μM) and sorafenib (5 μM) on PRP-dependent proliferation of hASCs (*n* = 7). **c** Effect of imatinib (5 μM) and sorafenib (5 μM) on PDGF-BB-dependent proliferation of hASCs (*n* = 7). **d** Anti-PDGF antibody inhibited PRP-stimulated proliferation of hASCs (*n* = 4). *P < 0.05. PDGF platelet-derived growth factor, PRP platelet-rich plasma

Cell proliferation of hASCs was also enhanced by treatment with 2 ng/ml PDGF-BB (*P* < 0.05 vs control). PDGF-BB at a concentration of 10 ng/ml markedly stimulated cell proliferation (*P* < 0.01 vs control and *P* < 0.01 vs 0.2% PRP). PDGF-BB displayed a dose-dependent stimulation of hASC proliferation between 0 and 10 ng/ml (Fig. 2a). Treatment with imatinib (5 μM) or sorafenib (5 μM) reduced the PRP-stimulated hASC proliferation (Fig. 2b). Similarly, both imatinib and sorafenib significantly inhibited the proliferation of hASCs induced by PDGF-BB (10 ng/ml). Inhibition with sorafenib was more potent than that with imatinib (Fig. 2c). Furthermore, treatment with anti-PDGF antibody inhibited PRP-stimulated growth of hASCs in a dose-dependent manner (Fig. 2d).

### PDGF-BB stimulated DNA synthesis

**Fig. 3:**
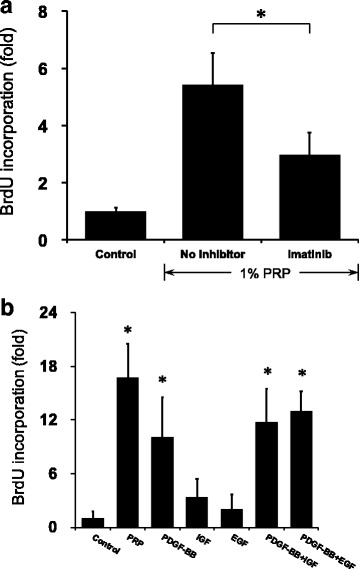
DNA synthesis in PRP-treated hASCs. Cells were incubated with PRP (1%) or PDGF-BB (10 ng/ml) in serum-free DMEM for 48 h. DNA synthesis measured by incorporation of BrdU. **a** Effect of PRP on DNA synthesis in hASCs (*n* = 7). **b** Effects of growth factors on stimulation of DNA synthesis in hASCs (*n* = 6). *P < 0.01. BrdU 5-bromo-2′-deoxyuridine, PRP platelet-rich plasma, PDGF platelet-derived growth factor, IGF insulin-like growth factor, EGF epidermal growth factor

Cell proliferation of hASCs was also evaluated using BrdU incorporation assays. Compared with control, PRP induced a 5.42-fold increase in BrdU incorporation, and imatinib decreased the incorporation 2.97-fold. Thus, PRP significantly stimulated DNA synthesis in hASCs, and imatinib inhibited DNA synthesis (Fig. 3a).

We also tested the stimulating effect of growth factors. PDGF-BB markedly enhanced DNA synthesis (10.08-fold vs control). In contrast, treatment with either IGF-I (10 ng/ml) (3.33-fold vs control) or EGF (10 ng/ml) (2.02-fold vs control) showed a minimal effect on DNA synthesis in hASCs. PDGF-BB plus either IGF or EGF stimulated DNA synthesis to a greater extent than IGF or EGF alone (Fig. 3b).

### Promotion of cell cycle transition from G0/G1 to S phase by PRP and PDGF-BB

**Fig. 4:**
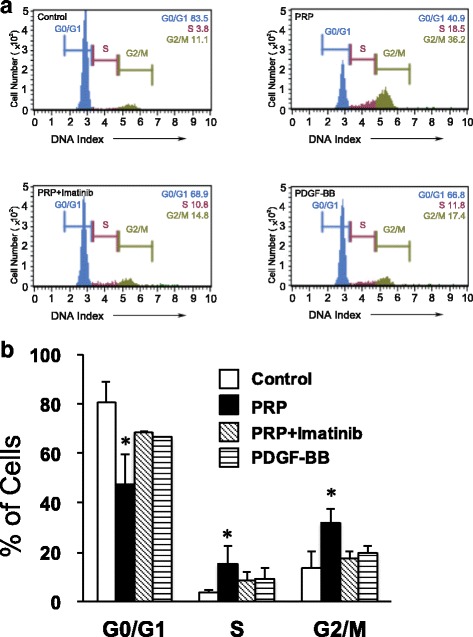
Analysis of the cell cycle in PRP-treated and PDGF-BB-treated hASCs. Cells were incubated with PRP (1%) or PDGF-BB (10 ng/ml) in serum-free DMEM for 48 h. Cell cycle stages determined by flow cytometry. **a** Representative data from four independent experiments. **b** Cell cycle distributions in hASCs after treatment with PRP and PDGF-BB (*n* = 4). *P < 0.01 compared with the control. PRP platelet-rich plasma, PDGF platelet-derived growth factor

When treated with PRP compared to control, the flow cytometry showed a trend in which cells in the S and G2/M phases increased (Fig. 4a). A histogram of the flow cytometry results is shown in Fig. 4b. The percent of cells treated with PRP that were in the S phase (15.44 ± 7.31%) was significantly higher compared with control (3.67 ± 0.91%). Similarly, the percent of cells treated with PRP that were in the G2/M phase (32.11 ± 5.5%) was also significantly higher compared with control (13.61 ± 6.63%). The percent of cells treated with PRP plus imatinib in the S and G2/M phases (8.81 ± 3.27% and 17.28 ± 3.15%, respectively) and the percent treated with PDGF-BB (9.01 ± 4.54% and 19.75 ± 2.97%, respectively) were not significantly different compared to control (3.67 ± 0.91% and 13.61 ± 6.63%, respectively). PRP stimulated cell progression to the S and G2/M phases, and imatinib inhibited this effect. PDGF-BB showed a small effect on the cell cycle (Fig. 4a, b).

### Activation of ERK1/2, Akt, and JNK signaling pathways with PRP and PDGF-BB

**Fig. 5:**
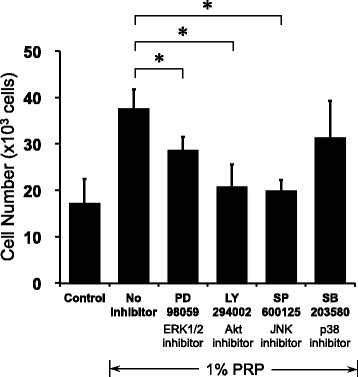
Pharmacological inhibition of PRP-induced proliferation through JNK and p38 pathways. After incubation in serum-free DMEM for 6 h, cells were treated with inhibitors at the designated concentrations for 48 h. Cell proliferation assessed with Cell Counting Kit-8. Cells treated with ERK1/2 inhibitor (PD98059, 20 μM), PI3K/Akt inhibitor (LY294002, 10 μM), JNK inhibitor (SP600125, 20 μM), or p38 inhibitor (SB203580, 10 μM) (*n* = 4). *P < 0.01 compared with no inhibitor. ERK extracellular signal-regulated kinase, JNK c-Jun N-terminal kinase, Akt protein kinase B, PRP platelet-rich plasma

To examine the signaling pathways involved in stimulation of hASCs by PRP, cells were treated with an ERK1/2 inhibitor (PD98059, 20 μM), PI3K/Akt inhibitor (LY294002, 10 μM), JNK inhibitor (SP600125, 20 μM), or p38 inhibitor (SB203580, 10 μM). PRP-induced cell proliferation was suppressed by PD98059, LY294002, and SP600125, but not SB203580 (Fig. 5).

**Fig. 6:**
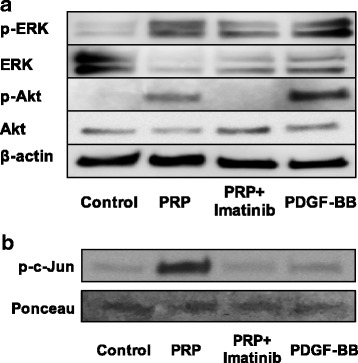
Phosphorylation of PRP-treated hASCs. After incubation in serum-free DMEM for 6 h, cells were stimulated with PRP (1%) or PDGF-BB (10 ng/ml) for 30 min. Imatinib added for 1 h before stimulation with PRP. **a** Immunoblots of p-Akt and p-ERK1/2. **b** JNK activity examined in cell lysates, c-Jun as the substrate. Amounts of c-Jun quantitated in comparison with Ponceau S staining (lower panel). ERK extracellular signal-regulated kinase, Akt protein kinase B, PRP platelet-rich plasma, PDGF platelet-derived growth factor

The signaling pathways activated by these treatments were further analyzed in hASCs with western blotting and a JNK activity assay. Phosphorylation of ERK1/2 and Akt increased following treatment with PRP or PDGF-BB. Imatinib inhibited the phosphorylation of these enzymes in the presence of PRP (Fig. 6a, b). Phosphorylation of JNK was not detected under these conditions. Next, we measured the activity of JNK using the substrate c-Jun. As shown in Fig. 6b, PRP markedly activated JNK. Thus, stimulation of cell growth by PRP was mediated through multiple signaling pathways.

## Discussion

We demonstrated that PRP enhanced the proliferation of hASCs through multiple signaling pathways by activating ERK1/2, JNK, and Akt. The proliferative effect of PRP, which was similar to the proliferative effect of PDGF-BB alone, was inhibited by the tyrosine kinase inhibitor imatinib and by the multikinase inhibitor sorafenib. The proliferative effect of PRP was also lowered by adding anti-PDGF antibody to the medium, indicating that PDGF-BB, which was abundant in activated PRP, played a major role in the proliferative effect of PRP. In addition, PRP induced the proliferation of cells in the S phase of the cell cycle, concomitant with an increase in BrdU uptake. Addition of PRP activated ERK, JNK, and Akt, and PRP-mediated proliferation was blocked by inhibitors of these signal transduction enzymes. On the other hand, PDGF-BB alone only slightly activated ERK, JNK, and Akt (Fig. 6a, b). These results indicated that other factors in PRP function in the additive effect on cell growth.

PRP is enriched in platelets, which were collected by centrifugation of autologous blood. Cytokines including PDGF, TGF-β, VEGF, IGF, EGF, and basic fibroblast growth factor (bFGF) are contained in α-granules of platelets. PRP was collected without coagulation, and was then activated by adding autologous thrombin and calcium chloride. Growth factors in activated PRP are indispensable for the proliferation of various types of cells [26]. We now demonstrated that PRP was a potent inducer of proliferation of hASCs.

Competence activity is known to be stimulated by factors that can make cells become “competent” to replicate their DNA and divide. Competence growth factors include PDGF [27] and FGF-2 [28]. PDGF and FGF-2 alone act on cells that are in either the G0 or early G1 phase of the cell cycle, rendering them competent to initiate DNA replication [28]. In contrast, progression activity refers to activity mediated by factors that can dictate the ultimate fraction of competent cells that enters the S phase [27]. These typical progression growth factors are EGF [28] and IGF-I [28]. The progression growth factors allow cells to progress through the pre-replicative phase of the cycle, inducing cells to enter the S, G2, and M phases. PRP comprises a large amount of competence growth factors, such as PDGF and FGF-2, and the progression growth factors, EGF and IGF-I. This study strongly suggests that these competence and progression growth factors act on hASCs in a concerted and compounding manner, and progress the cell cycle from the G0 phase to G1 and S phases.

Cell cycle progression is regulated by the expression of cyclins. Cyclins are factors that bind to and activate the cyclin-dependent kinases (CDKs). There are approximately 20 kinds of cyclins, such as cyclin A2, B1, and D1, and several types of CDKs such as CDK1, CDK2, and CDK4. These factors and kinases are known to control cell cycle progression by binding with each other in different combinations. Cyclin D is expressed in response to mitogens, and then binds with CDK4 or CDK6. The formed cyclin D complex phosphorylates the target protein, progressing the cell cycle from the G1 to the S phase. It was reported that the expression of cyclin D1 in hASCs increased with the transition of the cell cycle from the G1 to the S phase [24]. In addition, the cdc2/cyclin B complex was reported to regulate the G2/M phase transition [29]. This study found that the addition of PRP led to an increase in the proliferation of hASCs in the S and M phases, implying a possible involvement of cyclin D1 and B1.

The proliferative effect of PRP on preadipocytes [30], osteoblasts [31], and bone marrow mesenchymal stem cells [32] has been reported. Furthermore, PRP can enhance the proliferative effect of mesenchymal progenitor cells by activating the ERK signaling pathway, and PDGF-BB is a key factor in this stimulation [33]. Also, the proliferative effect on chondrocytes can be significantly stimulated by PRP via the ERK signaling pathway, and platelet-derived adenosine diphosphate in PRP is a key mediator of proliferation [34]. We found that PRP induces the proliferation of hDFs by the activation of ERK1/2 signaling [23]. Proliferation of hASCs induced by PRP was reported by Kakudo et al. [22] and Gersch et al. [35], but the signaling pathways remain unclear. PDGF-BB stimulates DNA synthesis in hASCs and cell proliferation, and these effects are mediated by JNK activation [24] or Akt activation [36]. Also, FGF-2 [37], EGF [38], or VEGF [39] induces the proliferation of hASCs through ERK1/2 activation. Our present study showed that the addition of PRP to hASCs activated JNK, ERK1/2, and Akt, and the addition of inhibitors of these kinases reduced the proliferative activity. Because PRP contains abundant PDGF-BB, FGF-2, EGF, and VEGF, the interaction among these growth factors may stimulate cell proliferation through multiple signaling pathways. The addition of PD98059, SP600125, or LY294002 to PRP-treated hASCs partially inhibited cell proliferation, which supports this conclusion. Hye Kim et al. [40] reported that an isoform of PDGF, PDGF-D, showed a strong proliferation effect on hASCs, and thus PDGF-D present in PRP may also induce proliferation of hASCs through the ERK1/2 and Akt pathways.

It is noted that the use of stem cells for therapeutic applications is influenced by their proliferative and differentiation potential, which is affected by the age of the donors. It was previously reported that compared with young cells, aged hASCs exhibited increased cellular senescence features [41–43], a decline in both stromal vascular fraction (SVF) cell yield [43] and hASC proliferation rate [41–44], a decreased differentiation potential [42] toward adipogenic [41, 43, 44], osteogenic [41, 43], and chondrogenic [41] lineages, negative effects on hASC frequency [43], fewer progenitor cell numbers [41], and impaired migration ability [43]. Based on these reports, we believe that hASCs obtained from older patients may have limitations in clinical application. Our study had limited sources of adipose acquisition due to the lack of younger patients, and thus it was impossible to compare the differences between young hASCs and aged hASCs. However, based on our experimental results, as proliferation of aged hASCs can still be stimulated by PRP, we speculate that young hASCs will have higher proliferative ability by PRP stimulation.

In future studies, we will use young hASCs to examine the effects of PRP on proliferation promotion.

## Conclusion

We found that both PRP and PDGF-BB can induce the proliferation of hASCs by activating ERK1/2, Akt, and JNK signaling pathways. This study clarified that PDGF-BB present in PRP plays an essential role in the proliferation via multiple signaling pathways, and is not limited to stimulation by PDGF-BB. The reason for the potent effect of PRP may be due to the presence of various factors involved in a variety of proliferative activities.

Thus, PRP is a powerful promoter to proliferate hASCs in vitro. Future studies are required to clarify the interaction of these factors that are present in PRP.
